# Genetic Predisposition to Solid Pediatric Cancers

**DOI:** 10.3389/fonc.2020.590033

**Published:** 2020-10-28

**Authors:** Mario Capasso, Annalaura Montella, Matilde Tirelli, Teresa Maiorino, Sueva Cantalupo, Achille Iolascon

**Affiliations:** ^1^Dipartimento di Medicina Molecolare e Biotecnologie Mediche, Università degli Studi di Napoli Federico II, Naples, Italy; ^2^CEINGE Biotecnologie Avanzate, Naples, Italy; ^3^European School of Molecular Medicine, Università Degli Studi di Milano, Milan, Italy

**Keywords:** genetic predisposition, germline variants, cancer predisposition genes, pediatric tumors, cancer susceptibility, germline-somatic interaction, SNP, next generation sequencing

## Abstract

Progresses over the past years have extensively improved our capacity to use genome-scale analyses—including high-density genotyping and exome and genome sequencing—to identify the genetic basis of pediatric tumors. In particular, exome sequencing has contributed to the evidence that about 10% of children and adolescents with tumors have germline genetic variants associated with cancer predisposition. In this review, we provide an overview of genetic variations predisposing to solid pediatric tumors (medulloblastoma, ependymoma, astrocytoma, neuroblastoma, retinoblastoma, Wilms tumor, osteosarcoma, rhabdomyosarcoma, and Ewing sarcoma) and outline the biological processes affected by the involved mutated genes. A careful description of the genetic basis underlying a large number of syndromes associated with an increased risk of pediatric cancer is also reported. We place particular emphasis on the emerging view that interactions between germline and somatic alterations are a key determinant of cancer development. We propose future research directions, which focus on the biological function of pediatric risk alleles and on the potential links between the germline genome and somatic changes. Finally, the importance of developing new molecular diagnostic tests including all the identified risk germline mutations and of considering the genetic predisposition in screening tests and novel therapies is emphasized.

## Introduction

Genomic sequencing studies have highlighted that pediatric cancers typically have few somatic mutations but a higher prevalence of germline alterations in cancer predisposition genes (1). The contribution of germline variants in pediatric tumors has been estimated between 8 and 12% (2, 3). Genetic variants are generally classified on the basis of their clinical effect: pathogenic variant means any sequence change that, differing from the consensus wild-type sequence, directly contributes to the development of the disease; likely pathogenic variants, instead, are genetic changes with a high likelihood of being disease-causing, but additional evidence is expected to confirm their clinical significance. Variant classification can arise from different methodologies and algorithms, which can assign different weights to collected data. However, studies cited in the present review generally refer to the American College of Medical Genetics and Genomics (ACMG) guidelines for variants interpretation (4). In this process, multiple categories of data (such as frequency in affected and unaffected populations, computational prediction tools, functional studies, and gene- or disease-specific information) are taken into account and combined to determine a variant pathogenicity classification.

It is also important to note that genetic variants can be detected through different genomic approaches and the type of identified alteration depends on the nature of the assay used. Large-scale genomic analyses such as whole-exome sequencing (WES) or whole-genome sequencing (WGS) can identify uncommon, moderate penetrant variants. Since WES investigates only the coding regions of the genome, it has proved very useful in detecting most of the causative variants of Mendelian diseases (5, 6). Furthermore, it has recently been used also to identify rare and uncommon causative mutations of complex diseases (7). On the other hand, WGS can capture nearly all known genetic variations, including those falling in regulatory elements, with much more uniform coverage of the genome, but it does not allow to detect mosaic variants with low clonality or variations causing DNA repetitions (8). Common, low-penetrance genetic variants, instead, are mostly identified by genome association study (GWAS), which assesses genotype–phenotype associations through testing of variants across genomes of many individuals, based on data obtained using numerous technologies, mostly WGS or genome-wide single-nucleotide polymorphism (SNP) arrays. Consequently, GWAS limitations are linked to the technology on which it is based: e.g., SNP array-based GWAS rely on pre-existing genetic variant reference panels (9). Finally, besides SNP array, copy-number variations (CNV) can be identified also through CGH array. Anyway, array methods cannot be used to detect single base pair changes, indels, balanced chromosome rearrangements, and low-percent mosaicism (10).

**Figure 1:**
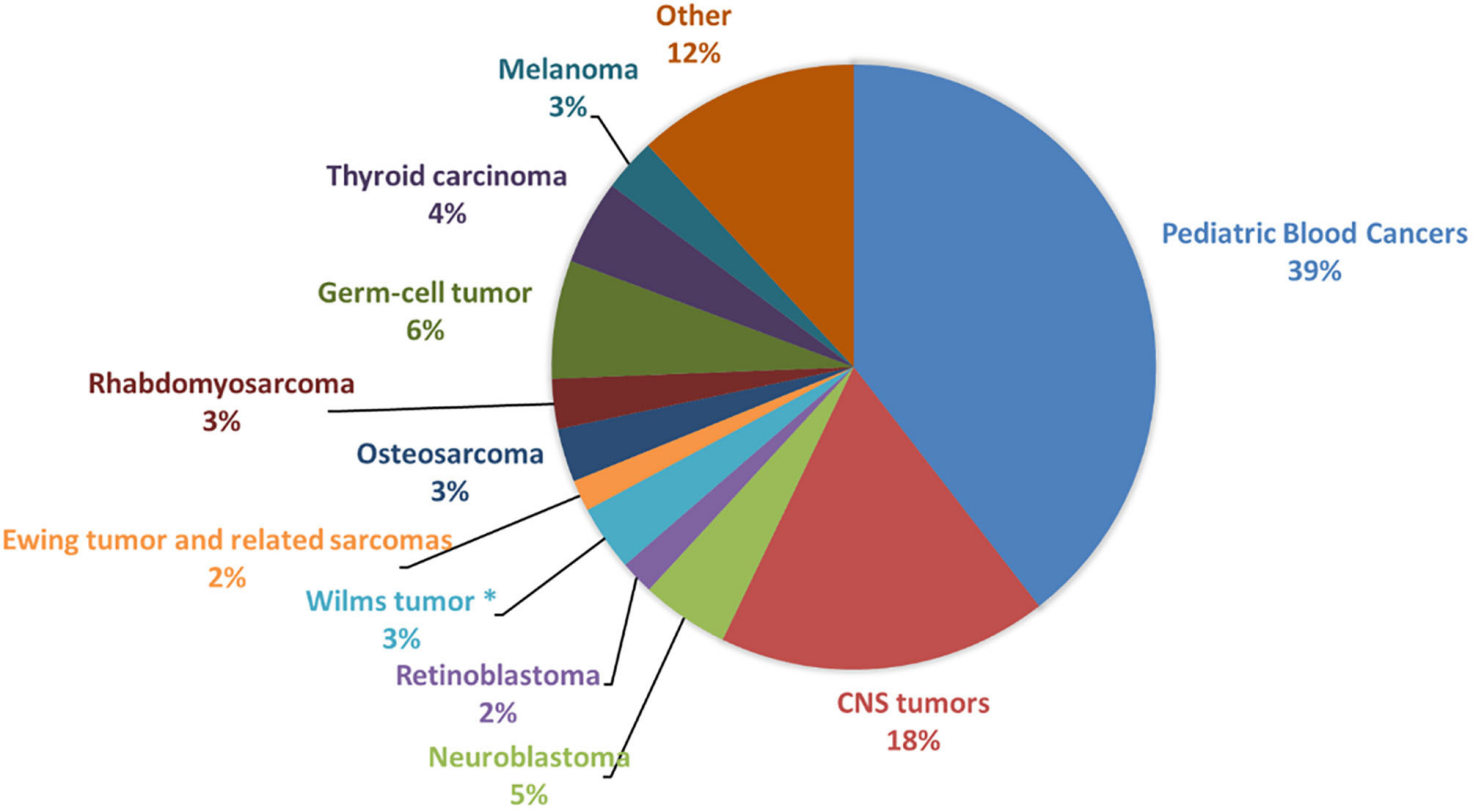
Frequency of pediatric cancers in patients younger than 19 years. The figure shows the prevalence of the main pediatric cancer types among patients younger than 19 years of age, calculated from Centers for Disease Control and Prevention (CDC) data (United States Cancer Statistics Data, https://wonder.cdc.gov/cancer.html) and based on incidence in United States for the years 1999–2016. CNS, Central Nervous System. *This frequency is related to Wilms tumor and other non-epithelial renal tumors.

Recently, in addition to germline pathogenic and/or likely pathogenic variants in known cancer-predisposing genes, it has been estimated that a high percentage (61%) of children, adolescent and young adult patients with solid tumors carry germline pathogenic and likely pathogenic variants in new candidate genes, including *PRKN, SMACAL1, SMAD7*, and *TMPRSS3* (3). The detection of cancer predisposition can lead to clinical benefits for patients, both for the molecular diagnosis and for the presence of specific biological features, as well as to eventually refine therapeutic choices. We provide an overview of the most significant knowledge of germline predisposition for the main pediatric solid tumors, which are central nervous system tumors (medulloblastoma, ependymoma and astrocytoma), neuroblastoma, retinoblastoma, Wilms tumor, osteosarcoma, rhabdomyosarcoma, and Ewing sarcoma, altogether accounting for 34.8% of all childhood cancers (Figure 1). Each tumor description is organized into two subsections: “familial cancer” and “sporadic tumor.” Familial cancer means a form of cancer that has higher incidence in families than in the general population due to rare, high-penetrance genetic variants. In this group, we also included rare genetic syndromes that are not usually considered as cancer syndromes but that predispose to the development of solid pediatric tumors. The second group, sporadic tumor, is referred to cancers which do not run in families and are intended as multifactorial diseases whose onset can be attributed to the combined effect of environmental and genetic factors. In sporadic cancers, genetic factors can be categorized into two types: uncommon, moderate-penetrance genetic variants, which for the studies considered in this review show a frequency lower than 1–0.001% in the general population and are not so rare as those associated with familial cancer, and common, low-penetrance genetic variants.

The knowledge of genetic mutations responsible for syndromic disorders associated with the risk of developing pediatric cancer has greatly increased over the past years (11). Indeed, several tumor predisposing syndromes are the underlying cause of at least 8.5% of cancers in pediatric patients (12). Thus, the role of general practitioners and pediatricians in recognizing the major cancer genetic-associated syndromes, in making appropriate referrals for genetic counseling and testing when indicated, is crucial for a specific monitoring and management of the patient.

Most cancer susceptibility genes are involved in fundamental biological pathways such as cell-cycle control, chromatin remodeling, or DNA repair. Therefore, alterations in these genes compromise the normal control of cell growth and lead to a substantial increase in the risk of developing cancer. Another element of great interest discussed here is the presence of cooperation between germline and somatic alterations, which can represent an early tool for evaluating the clinical outcome and for the stratification of patients in risk subgroups. We also discuss evidence that points to a need for more collaborative investigations in identifying driver events in pediatric cancers.

## Central Nervous System Tumors

**Figure 2:**
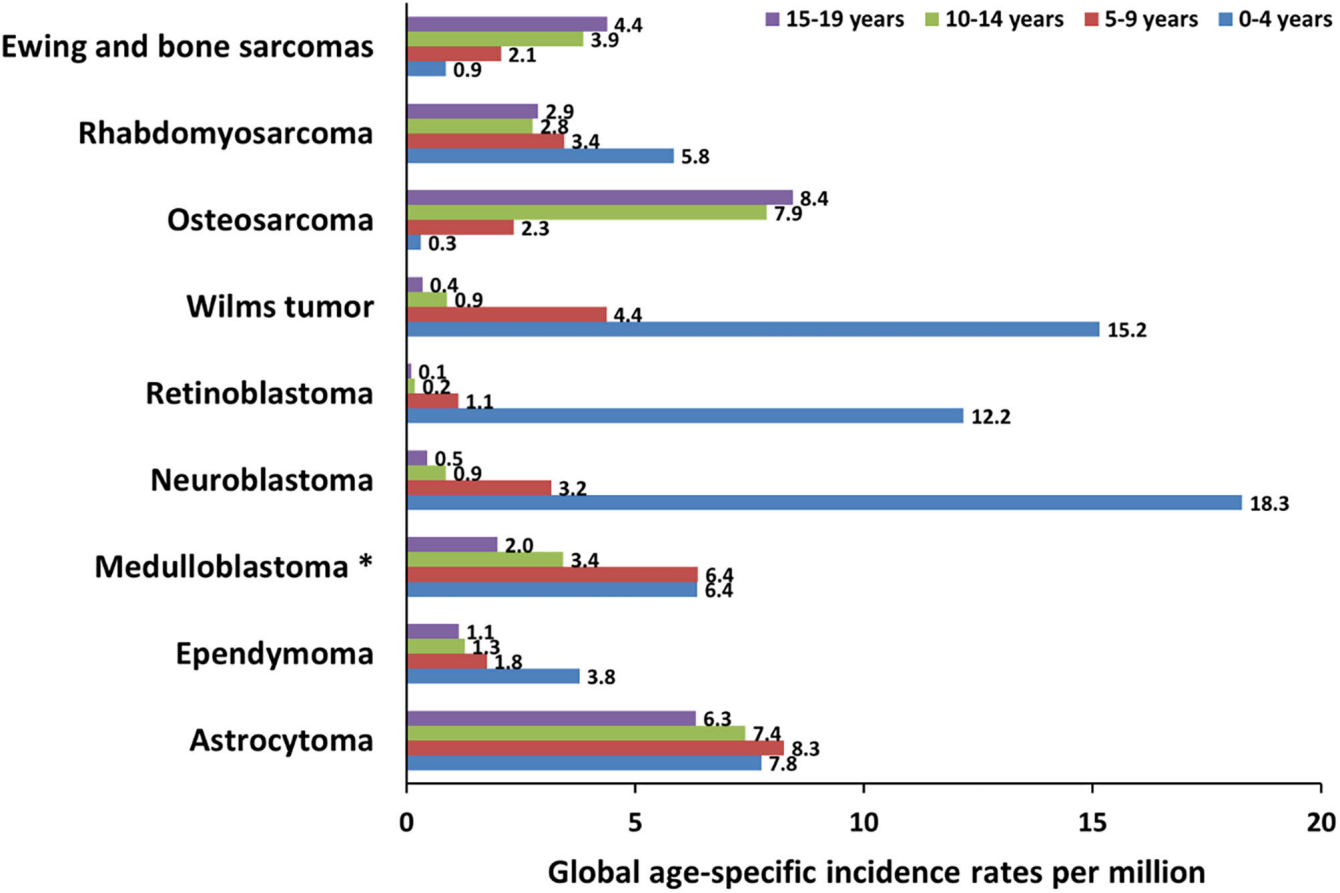
Global incidence of pediatric cancers in patients younger than 19 years. The graph shows the global age-specific incidence rates (ASR) per million for individual age groups (0–4 years, 5–9 years, 10–14 years, and 15–19 years) of pediatric cancer types discussed in this review. ASR reported next to the bars are calculated from International Incidence of Childhood Cancer (IICC, https://iicc.iarc.fr/) data. *These ASR include also less frequent embryonal central nervous system tumors.

Central nervous system (CNS) tumors represent the most frequent types of cancer in children aged 0–14 years, with a mortality rate of 0.72 per 100,000 population (13). The three most frequent tumors are medulloblastoma (MB), ependymoma (EP), and astrocytoma (AS) (Figure 2).

### Medulloblastoma

MB is an embryonal tumor of cerebellum (14) that affects children under the age of 14, with an average onset of about 6–8 years (Figure 2) and with a 5-year overall survival for standard-risk patients of 70–85% (14). It is classified into four genetic and molecular groups: the first two groups, WNT-activated (MB_WNT_) and Sonic Hedgehog activated (MB_SHH_), are named for the signaling pathways that play prominent roles in the pathogenesis of those subgroups, while, since less is known about the biology of the remaining two subgroups, they are numerically designated as “Group 3” and “Group 4” (14). Damaging germline mutations in known cancer-predisposing genes play an important role in two main subgroups, MB_WNT_ and MB_SHH_, in which genetic testing is highly recommended (15). MB_WNT_ is characterized at somatic level by activating mutations in exon 3 of β-catenin (*CTNNB1*) and monosomy of chromosome 6, while MB_SHH_ by amplification of *GLI2* and *MYCN*, as well as loss of 17p (16).

#### Familial Medulloblastoma

To date, only germline mutations in *ELP1* have been found in two independent families with MB_SHH_ (17). Although inherited or familial MB is extremely rare, there are few rare inherited syndromes that are associated with increased risk of developing this tumor (**Table 2**). Germline mutations of *PTCH1* and *SUFU*, by causing activation of the SHH signaling pathway, predispose to MB_SHH_ in Gorlin syndrome, an autosomal dominant disease caused by mutations in *PTCH1* (67, 124). In Turcot syndrome, a rare disorder characterized by the association of colonic polyposis and primary brain tumors, germline mutations of *APC* predispose to the development of MB_WNT_ (114). In MB_WNT_, activation of the WNT pathway is due to somatic mutations of *CTNNB1* in most of tumors but it is also observed in patients with only germline mutations of *APC*, stressing the importance of genetic predisposition in high-risk patients (15, 114). Germline mutations in *BRCA2* and *PALB2*, associated or not associated with Fanconi anemia, have been found in MB_SHH_ (58, 125) and are often observed in association with somatic homologous recombination repair defects (15). The role of germline mutations in *TP53* in MB is still widely debated today. *TP53* germline mutations affect MB prognosis differently according to the different subgroups: germline mutations in MB_SHH_ are associated with poor prognosis, while both germline and somatic mutations in MB_WNT_ are associated with better prognosis. This may be due to a different origin of the MB itself (14). Patients with germline *TP53* mutations can have tumors characterized by catastrophic DNA chromothripsis and are often associated with Li–Fraumeni syndrome (LFS), a cancer predisposition disorder caused by germline mutations of the tumor-suppressor p53 (71). Other MB-associated syndromes are Bloom's syndrome (31), ataxia telangiectasia (18), and Greig's cephalopolysyndactyly syndrome (14, 40, 45, 85, 122) (**Table 2**).

#### Sporadic Medulloblastoma

The association between MB and genetic syndromes explains most of the genetic predisposition to MB. However, sporadic forms are known in literature and are partially explained through uncommon, moderate penetrant mutations identified by whole-exome sequencing (WES) or whole-genome sequencing (WGS), or common, low-penetrance genetic variants identified by genome wide association study (GWAS) (Table 1 and **Table 3**).

**Table 1:** Rare, high-penetrance, and uncommon, moderate-penetrance variants in genes predisposing to pediatric tumors and main biological pathways.

**Pathways**	**Gene(s)**	**Tumors**	**References**
Collagen chain polymerization	*COL7A1*	NB, RMS, WT	(3)
Cytoskeletal and adhesion signaling	*GJB2*	AS, CNS tumors, EWS, OS, RMS	(3, 126)
	*CDH1*	WT	(3)
DNA base excision repair (BER)	*ERCC2*	AS, OS	(127–129)
DNA double-strand break repair (DSB)	*BRCA1*	AS, CNS tumors, EWS, OS, RB	(3, 126, 129, 130)
	*BRCA2*	AS, NB, MB, RMS	(2, 3, 15, 58, 125, 126)
	*CHEK2*	CNS tumors, EWS, NB, OS, RB, RMS, WT	(3, 129, 131, 132)
	*BAP1*	RB	(3)
	*BLM*	EWS, MB	(15, 130)
	*BRIP1*	EWS, MB, OS	(2, 3, 15, 129, 130)
	*NBN*	MB	(15)
	*WRN*	MB	(15)
	*PALB2*	MB, OS, WT	(3, 15, 129, 131, 132)
DNA mismatch repair system (MMR)	*MSH2*	WT, OS	(2, 3)
	*MSH6*	RB, RMS, WT	(3, 133)
	*PMS2*	AS, CNS tumors, EWS	(2, 3, 127, 130)
DNA repair	*FANCA*	AS, MB	(15, 126)
	*FANCC*	EWS, MB	(2, 15, 130)
	*FANCI*	RMS	(133)
	*FANCL*	OS	(2, 129)
	*FANCM*	OS	(2, 129)
	*ATR*	RMS	(3)
	*MUTYH*	AS, EWS	(2, 127)
	*RAD51D*	WT	(3)
	*RECQL4*	OS	(129)
Genome stability and regulation of cell cycle	*ALK*	Familial/sporadic NB	(2, 3, 134, 135)
	*ATM*	EWS, MB, OS, RB, RMS	(3, 15, 129, 133)
	*RB1*	OS, familial/sporadic RB	(2, 3, 129, 135, 136)
	*TP53*	AS, EWS, MB, NB, OS, RMS, WT	(2, 3, 15, 127, 129–131, 133, 135, 137–139)
Metabolic pathways	*HMBS*	CNS tumors	(3)
	*FAH*	OS	(129)
	*SDHA*	NB	(3)
Protein interaction at synapsis	*PTPRD*	Advanced/metastatic EWS	(140)
Protein translation and modification	*KIF1Bβ*	Familial NB	(141)
RET signaling and G-protein signaling, H-RAS regulation pathway	*ERBB4*	NB	(3)
	*NF1*	AS	(126)
	*RET*	EWS	(2, 130)
miRNA processing genes	*DIS3L2*	WT	(131, 132, 137)
	*DROSHA*	WT	(131, 137)
	*XPO5*	WT	(131)
	*DICER1*	Familial/sporadic WT, RMS	(3, 52, 55, 131, 137, 142)
Sonic Hedgehog pathway (SHH)	*GPR161*	MB	(143)
	*PTCH1*	MB	(15, 67)
	*SUFU*	MB	(15, 67)
Spindle assembly checkpoint (SAC)	*TRIP13*	Familial WT	(83)
Transcriptional regulation and chromatin remodeling	*CTR9*	Familial WT	(144)
	*ELP1*	MB	(17)
	*LZTR1*	CNS tumors, EWS	(3)
	*PHOX2B*	Familial NB	(145)
	*POLE*	EWS, NB	(3, 130)
	*SMARCA4*	NB	(3, 146)
	*REST*	Familial/sporadic WT	(147, 148)
	*TRIM28*	Familial/sporadic WT	(147)
	*WT1*	Familial/sporadic WT	(147, 149, 150)
WNT signaling pathway	*APC*	MB	(15)
Other	11p15	Familial/sporadic WT	(150, 151)

*Rare, high-penetrance variants are related to familial forms of tumors, while uncommon, moderate-penetrance variants refer to sporadic forms. When the tumor form is not specified we refer to uncommon, moderate-penetrance variants. AS, astrocytoma; CNS, central nervous system; EP, ependymoma; EWS, Ewing sarcoma; MB, medulloblastoma; NB, neuroblastoma; OS, osteosarcoma; RB, retinoblastoma; RMS, rhabdomyosarcoma; WT, Wilms tumor*.

#### Uncommon, Moderate-Penetrance Variants

In a study on 1,022 MB patients, novel partial or total *APC* deletions were found (15). These mutations were not associated with any familial syndrome and predisposed to MB_WNT_. In the same study, 1% of patients (classified as MB_SHH_) had *TP53* mutations but only 5/11 patients showed family history of cancer, emphasizing the role of *TP53* germline mutations in predisposing to sporadic MB. Notably, germline missense, frameshift, or non-sense mutations in the DNA-binding domain of *TP53* were found to be associated with a series of events at the somatic level such as rearrangements, chromothripsis, and loss of heterozygosity in MB_SHH_ patients, whereas germline mutations in *SUFU* and *PTCH1* co-occurred with somatic loss of heterozygosity (15) (**Table 4**). These results further provide evidence that novel associations between germline variants and specific somatic events, beyond those reported by Knudson in 1971, can play a role in carcinogenesis. Indeed, recent body of literature supports the hypothesis that specific germline variants determine which somatic events and mutations are generated and selected in cancer cells during tumorigenesis (179).

MB can also arise in patients with germline mutations in other known cancer genes such as *ATM, FANCA, FANCC, NBN, WRN, BLM*, and *BRIP1* and in candidate genes like *CHEK2, CREBBP, RAD51, ERCC2*, and *ERCC4*. All of these genes are involved in cell-cycle regulation and DNA repair (15). Frameshift, protein-truncating, and missense mutations occurring in *GPR161*, a gene never previously associated with MB, were found in 6 MB_SHH_ cases (143) that, at the somatic level, showed loss of heterozygosity with retention of the mutated allele, confirming its role as driver gene in MB_SHH_. *GPR161* functions are essential for embryonic development and for the proliferation of granular cells (143). Germline mutations in *ELP1* have been very recently found to predispose to MB_SHH_ and to be associated with two consecutive somatic events: loss of the 9q arm, with consequent loss of the wild-type copy of *PTCH1* and *ELP1*, and a second independent mutation event in *PTCH1* (17) (**Table 4**). This study, importantly, showed that 40% of MB_SHH_ patients carry disease-predisposing mutations and that genetic predisposition to proteome instability may be a determinant in the pathogenesis of pediatric brain cancers (17) (Table 1).

#### Common, Low-Penetrance Variants

To date, there are no relevant GWAS conducted to identify common variants associated with MB. Only one study has been performed in a small sample including 244 MB cases and 247 control subjects from Sweden and Denmark, but no locus reached the significance threshold (154). The most significant locus was 18p11.23 including *PTPRM* (154). A different approach that starts from the most frequently mutated genes in MB such as *CCND2, CTNNB1, DDX3X, GLI2, SMARCA4, MYC, MYCN, PTCH1, TP53*, and *KMT2D* was proposed to identify MB-associated common variants (162). Eight variants, located in *CCND2, PTCH1*, and *GLI2*, associated with the risk of developing MB (162) (**Table 3**). However, these findings need further validation in independent cohorts of cases and controls.

Microsatellites are tandem repeats of 1–6 base pairs, and their variability is associated with numerous tumors, including MB. In a recent work, starting from WES and WGS data, the authors developed an algorithm able to identify a signature of 43 microsatellites that distinguished with high-sensitivity and specificity MB subjects from controls in two independent sets of MB cases and controls (180). Interestingly, *in silico* analyses revealed that genes harboring these microsatellite loci had cellular functions important for tumorigenesis (180).

### Other Brain Tumors

EP originates from the walls of the ventricular system (79), arises between 0 and 4 years (Figure 2) (79), and has a 5-year overall survival of about 60% (181). EP is diagnosed in ~33–53% of patients with type 2 neurofibromatosis, with high occurrence of truncating mutations in *NF2* (97). EP has recently been associated with Kabuki syndrome, with mutations in *KMT2D* (70) and rarely occurs in Turcot and MEN1 syndromes with mutations in *MSH2* and *MEN1*, respectively (79) (Table 2). To date, large studies on common variants and sporadic forms are lacking (Table 1). AS is classified into several forms including pilocytic, anaplastic, diffuse, and glioblastoma (182). Pilocytic AS is the most common form in children and young adults, with an average age at onset between 0 and 9 years (13) (Figure 2) and a 5-year survival of 94.1% (13). Regarding the genetic predisposition, one large study reported germline splicing mutations in the tumor-suppressor genes *MUTYH* and *ERCC2* and point mutations in *TP53* and *PMS2* (127) (Table 1). Pathogenic mutations in *NF1, BRCA2, FANCA*, and *GJB2* have been also identified in a recent study involving 280 patients with different forms of AS (126).

**Table 2:** Syndromes associated with pediatric tumors. Frequencies reported refer to the occurrence rate of pediatric cancers in patients with genetic syndromes.

**Syndrome/disease**	**Inheritance pattern**	**Gene/s associated**	**Tumor**	**Frequency**	**References**
Ataxia telangiectasia	AR	*ATM*	MB	Extremely rare	(18)
ATR-X syndrome	AR	*ATR-X*	OS	Extremely rare	(19)
Baller–Gerold syndrome	AR	*RECQL4*	OS	Extremely rare	(20, 21)
Beckwith–Wiedemann syndrome	Imprinting, AD	*CDKN1C*	NB	4–21%	(22, 23)
		*KCNQ1OT1*	RMS	7.5%	(24–28)
		*11p15 or H19 loci*	WT	7–30%/20%	(29, 30)
Bloom syndrome	AR	*RECQL3 (BLM)*	MB	Extremely rare	(31)
			OS	2%	(32, 33)
			WT	<5%	(29, 34)
Bohring-Opitz syndrome	AD	*ASXL1*	WT	7%	(35, 36)
CCHS/hirschsprung syndrome	AD	*PHOX2B*	NB	10–20%	(37–39)
Constitutional mismatch repair deficiency	AR	*MSH2, MSH6, MLH1, PMS2*	MB	11.6%	(33, 40)
Costello syndrome	AD	*HRAS*	NB	17%	(41)
			RMS	17%	(42–44)
Curry–Jones syndrome	Unknown	*GLI3*	MB	Extremely rare	(45, 46)
Diamond–Blackfan anemia	AD	*Unknown*	OS	<1%	(33, 47–50)
Denys–Drash syndrome	AD	*WT1*	WT	90%	(51)
DICER1 syndromes	AD	*DICER1*	RMS	Rare	(52–54)
			WT	<5%	(29, 55)
Familial paraganglioma/pheochromocytoma syndrome	AD	*SDHB*	NB	Rare	(56)
Fanconi anemia	AR	*BRIP1, BRCA2, PALB2*	NB	rare	(57)
		*BRCA2, PALB2*	MB,	25%	(58, 59)
			WT	>20%	(60–62)
Frasier syndrome	AD	*WT1*	WT	5–10%	(63)
Gorlin syndrome	AD	*PTCH1*	RMS	Rare	(64, 65)
			WT	<5%	(36, 65, 66)
		*PTCH1*	MB	<2%	(67, 68)
		*SUFU*		30-40%	
Hyperparathyroidism-jaw tumor syndrome	AD	*CDC73 (HRPT2)*	WT	<5%	(60)
Isolated hemihypertrophy	AD	*11p15 locus*	WT	6%/ <5%	(69)
Kabuki syndrome	AD	*KMT2D*	EP	Extremely rare	(70)
Li–Fraumeni syndrome	AD	*TP53*	MB	14%	(68, 71)
			NB	rare	(72)
			OS	12%	(73–76)
			RMS	80%	(75, 77)
			WT	<5%	(29, 78)
MEN1 syndrome	AD	*MEN1*	EP	Rare	(79)
Mosaic variegated aneuploidy syndrome	AR	*BUB1B*	RMS	High	(80, 81)
		*BUB1B, TRIP13*	WT	>20%	(60, 80, 82, 83)
Muliebry nanism syndrome	AR	*TRIM37*	WT	<5%	(29, 84)
Nijmegen breakage syndrome	AR	*NBN*	MB	Extremely rare	(85)
		*NBS1*	RMS	Rare	(86, 87)
Noonan syndrome	AD	*PTPN11, KRAS*	NB	17%	(88)
		*SOS1*	RMS	Rare	(89–93)
Noonan-like syndrome	AD	*CBL*	RMS	Extremely rare	(94)
Neurofibromatosis type I	AD	*NF1*	NB	Rare	(95, 96)
			RMS	0.5%	(44)
Neurofibromatosis type II	AD	*NF2*	EP	3–6%	(68, 97)
Paget's disease of bone	AD	*Unknown*	OS	<1%	(98, 99)
Perlman syndrome	AR	*DIS3L2*	WT	50–60%	(33, 100)
PIK3CA-related segmental overgrowth	Unknown	*PIK3CA*	WT	<5%	(29, 101)
ROHHAD	Unknown	*Unknown*	NB	Rare	(39)
Rothmund–Thomson and RAPADILINO syndrome	AR	*RECQL4*	OS	30–60%, 13.3%	(33, 102–108)
Rubinstein–Taybi syndrome	AD	*CREBBP, P300*	MB	Extremely rare	(14)
		*CREBBP*	NB	Extremely rare	(77, 109)
Simpson–Golabi–Behmel syndrome	X-linked	*GPC3*	NB	10%	(77)
			WT	10%	(60, 82, 110)
Sotos syndrome	AD	*NSD1*	NB	Rare	(111, 112)
			WT	<5%	(36, 113)
Turcot syndrome	AR	*APC*	MB	<1%	(68, 114)
		*MSH2*	EP	53%	(68, 79)
WAGR syndrome	AD	*WT1*	WT	50%	(60, 115)
Weaver syndrome	AD	*EZH2*	NB	Rare	(116, 117)
Werner syndrome	AR	*RECQL2 (WRN)*	OS	7%	(108, 118–120)
Wolf–Hirschhorn syndrome	Unknown	*MSX1*	NB	Extremely rare	(121)
Xeroderma pigmentosum	AR	*DDB2, ERCC1, ERCC2, ERCC3, ERCC4, ERCC5, POLH, XPA, XPC*	MB	Extremely rare	(122)
13q deletion syndrome	Unknown	*RB1*	RB	Variable	(123)

*AD, autosomal dominant; AR, autosomal recessive; EP, ependymoma; MB, medulloblastoma; NB, neuroblastoma; OS, osteosarcoma; RB, retinoblastoma; RMS, rhabdomyosarcoma; WT, Wilms tumor*.

## Neuroblastoma

Neuroblastoma (NB) originates from neural crest cells and affects the nervous sympathetic system (183). NB exhibits unique features, such as early age of onset, high frequency of metastatic disease at diagnosis in patients over 1 year of age (Figure 2), and the tendency for spontaneous regression of tumors in infants. In high-risk cases, the survival rate is only 50% (183). NB tumors, as well as other pediatric cancers, present few recurrent somatic mutations but frequent chromosomic aberrations such *MYCN* amplification, 17q gain, 1p deletion, and 11q deletion (184).

### Familial Neuroblastoma

Familial NB represents 1–2% of cases, with *PHOX2B* and *ALK* as major susceptibility genes (184) (Table 1). The first identified familial gene is *PHOX2B* (37, 145), already associated with congenital central hypoventilation syndrome (CCHS) (185) and encoding a transcription factor driving neural crest differentiation (186). NB-exclusive mutations are mainly missense and frameshift (187). *PHOX2B* germline mutations account for ~10% of familial NB (188), but this gene is also mutated in 2% of sporadic cases (189). Subsequently, the major susceptibility gene was identified in *ALK*. Its gain-of-function mutations, which account for 75% of familial cases (134, 188), are mainly located in the kinase domain of the encoded tyrosine kinase receptor and show incomplete penetrance (190). *ALK* somatic mutations are also reported in 10–12% of primary sporadic NB tumors (134, 191). Additional NB-predisposing genes have not yet been discovered. Mutations in *KIF1B*β (141) and *GALNT14* (192) and in 16p12–13, 4p16, and 1p loci (193–195) (Table 1) have been reported in related patients, but further validations are needed.

Children suffering from specific cancer predisposition syndromes such as LFS and others (Table 2) show an increased NB risk (22, 38, 39, 41, 56, 57, 72, 77, 88, 95, 111, 116, 121). Thus, protocols for NB surveillance need to be established.

### Sporadic Neuroblastoma

Only a small subset of sporadic NB cases has an identifiable somatic oncogenic point mutation (196, 197), suggesting that predisposing genetic factors found in GWAS studies could cooperate to increase disease occurrence (198, 199).

#### Uncommon, Moderate-Penetrance Variants

Recent studies focused on uncommon germline variants, which presumably have a larger effect on predisposition compared to common ones. In different studies, pathogenic and likely pathogenic variants were identified in predisposition genes such as *ALK, CHEK2, BRCA2, SMARCA4*, and *TP53* (Table 1) but also in candidate genes like *AXIN2, PALB2, BARD1, PINK1, APC, BRCA1, SDHB*, and *LZTR1* (2, 135, 146, 196, 197, 200) Specifically, *TP53* variants are strongly associated with NB susceptibility (201). All the mentioned genes are involved in DNA repair and maintenance of genomic integrity (Table 1).

#### Common, Low-Penetrance Variants

GWAS studies identified several NB susceptibility loci (Table 3) including *CASC15* (160), *BARD1* (157), *LMO1* (175), *HACE1*, and *LIN28B* (155) associated with high-risk NB, whereas *DUSP12, HSD17B12, DDX4*, and *IL31RA* associated with the low-risk NB group (161, 198). Functional studies of these loci have highlighted the key role of GWAS in elucidating NB carcinogenesis. A SNP in the long non-coding RNA (lcnRNA) *CASC15* produces a truncated isoform, whose lower expression correlates with advanced disease (202). Loss of another lncRNA, *NBAT-1*, at the same locus, contributes to aggressive NB by increasing proliferation and impairing differentiation of neuronal precursors (203). Diverse functional studies have elucidated the role of *BARD1* and its variants in NB development (204). Variants in the *BARD1* promoter decrease the expression of the tumor-suppressor form which protects NB cells from DNA damage (205, 206), whereas variants in introns increase the expression of an oncogenic isoform, *BARD1*β, which stabilizes the Aurora kinases (207, 208). *LMO1* decreased expression, caused by a variant in a super-enhancer which disrupts GATA binding (209), reduces NB cell proliferation. Finally, the activation of *LIN28B*, due to genetic variants, can enhance MYCN levels via let-7 microRNA suppression (155, 210, 211). The genetic landscape of sporadic NB has been amplified with the discovery of additional susceptibility genes including *RSRC1*/*MLF1* and *CPZ* (159), *SPAG16* (177), *NEFL* (156), and *CDKN1B* (170).

**Table 3:** Common, low-penetrance variants in genes predisposing to pediatric tumors and main biological pathways.

**Pathways**	**Gene(s)**	**Tumors**	**References**
Centrosome stabilization	*KIZ*	EWS	(152)
Cytoskeletal and adhesion signaling	*NHS*	WT	(153)
	*PTPRM*	MB	(154)
Differentiation	*NKX2-2*	EWS	(152)
	*NEFL, LIN28B*	NB	(155, 156)
DNA double-strand break repair (DSB)	*BARD1*	NB, WT	(157, 158)
Extracellular matrix remodeling	*MMP20*	NB	(159)
Genome stability and regulation of cell cycle	*BMF*	EWS	(152)
	*CASC15/NBAT-1, DUSP12*	NB	(160, 161)
	*CCND2*	MB	(162)
	*MDM2, MDM4*	RB	(163, 164)
Immunity pathways	*HACE1, IL31RA*	NB	(155, 161)
Metabolic pathways	*ACYP2*	OS	(165, 166)
	*HSD17B12*	NB	(161)
	*PCSK9, TCN2*	WT	(153)
Protein translation and modification	*CPZ, DDX4, KIF1*	NB	(159, 161, 167, 168)
	*DDX3X*	MB	(162)
Replication and telomere maintenance	*TERC, NAF1, TERT, OBFC1, CTC1, RTEL1*	OS	(165, 166)
RET, RAS, and G-proteins signaling	*CDKN1A*	RB	(169)
	*CDKN1B*	NB	(170)
	*KRAS*	WT	(171)
RNA biogenesis and processing	*DDX1*	WT	(153)
	*TARDBP*	EWS	(172)
Sonic Hedgehog pathway (SHH)	*GLI2*	MB	(162)
Synaptic proteins and neurotransmitters	*DLG2*	WT	(153)
	*GRM4*	OS	(173)
Transcriptional regulation and chromatin remodeling	*EGR2, NR0B1, RREB1*	EWS	(152, 172, 174)
	*KMT2D, MYC, MYCN, SMARCA4*	MB	(162)
	*LMO1, RSRC1/MLF1*	NB	(159, 175)
	*NFIB*	Metastatic OS	(176)
WNT signaling pathway	*CTNNB1*	MB	(162)
Others	2p25.2	OS	(173)
	*SPAG16*	NB	(177)

*EWS, Ewing sarcoma; MB, medulloblastoma; NB, neuroblastoma; OS, osteosarcoma; RB, retinoblastoma; WT, Wilms tumor*.

Reanalyses of GWAS data have discovered novel mechanisms and genetic factors that promote NB development (Table 3). Two studies clearly demonstrate a cooperation between predisposing variants and somatic aberrations in NB initiation (Table 4). Indeed, SNPs in *MMP20* (167) and *KIF15* (168) increase NB susceptibility in the presence of 11q deletion and *MYCN* amplification, respectively, whereas another study shows that specific mtDNA haplogroups can influence the risk of NB (212). We have provided evidence that SNPs in *PARP1* and *IL6* might be predictive biomarkers of response to chemotherapy and prognosis (213, 214). Finally, our recent works found that NB shares risk loci with other complex diseases and tumors. Indeed, SNPs in 2q35, 3q25.32, and 4p16.2 are cross-associated with congenital heart disease (CHD) and NB (215), while 1p13.2 showed cross-association with NB and melanoma (216). Very recently, a cross-match investigation between germline alterations in pediatric patients with different solid tumors and CHD-related genes has identified that NB is among the tumors with the highest enrichment of germline pathogenic and likely pathogenic variants in these genes (3).

**Table 4:** Germline–somatic interactions identified in genes predisposing to pediatric tumors.

**Tumors**	**Gene**	**Frequency**	**Somatic interaction**	**References**
MB	*TP53*	Rare	DNA chromothripsis	(71)
	*ELP1*	Rare	Loss of the 9q arm and a second independent mutation event in *PTCH1*	(17)
NB	*KIF15*	Common	Increased NB risk in presence of *MYCN* amplification	(168)
	*MMP20*	Common	Increased NB risk in presence of 11q deletion	(167)
EWS	*EGR2*	Common	EWSR1-FLI1 chimera	(178)
	*NR0B1*	Common		(174)

*EWS, Ewing sarcoma; MB, medulloblastoma; NB, neuroblastoma*.

### Constitutional Chromosomal Abnormalities

Highly associated with NB are hemizygous deletion in 1q21.1, disruption in *NBPF23* (217), and microdeletion in 16p11.2, containing *SEZ6L2* and *PRRT2* (218). Deletion including *SLFN11*, duplication of *SOX4*, and partial deletion of *PARK2* have been identified in three different patients, respectively (219).

## Retinoblastoma

Retinoblastoma (RB) is a pediatric malignancy of the neural retina, commonly initiated by biallelic inactivation of *RB1* (220) and affecting one (unilateral) or both eyes (bilateral). The median age at diagnosis is 12 months in bilateral tumors and 24 months in unilateral ones (220) (Figure 2). Patient survival is >95% in high-income countries but <30% globally (220). The first studies on RB unveiled the importance of genetics in cancer; indeed, the “two-hit hypothesis” formulated by Knudson (221) on *RB1* has been paradigmatic for the understanding of tumor-suppressor genes and the study of familial cancers.

### Familial Retinoblastoma

Hereditary RB encompasses about 40% of all cases with most having bilateral tumors, 15% unilateral, and 5% trilateral (associated with midline brain tumor) (220). Familial RB is distinctly associated with the *RB1* tumor-suppressor gene, which encodes pRB, a crucial regulator of the cell cycle. Germline mutations in *RB1* are inherited in 25% of cases in an autosomal-dominant manner. A broad spectrum of inactivating *RB1* germline mutations have been described, mainly nonsense and frameshifts affecting the coding region, few large deletions, and <5% silencing gene promoter (136). Penetrance and expressivity can vary within families due to partially functional *RB1* alleles (222, 223) or parent-of-origin effect (224). Influence of genetic modifiers such as *MDM2, MDM4* (225, 226), or *MED4* (227) and polymorphisms in p53 (228), *CDKN1A* (169), and *CDKN2A* (229) could also influence RB development. Reduced *MDM2* and *MDM4* expression may increase the *RB1* haploinsufficiency, whereas variants affecting the activity of p53 pathway effectors impact cell-cycle arrest. However, studies on larger cohorts of patients are required to confirm these findings. A small subset of hereditary RB patients is not carrier of *RB1* mutations. Investigation through a clinical exome gene panel within 3 families proposed *FGFR4, NQO1, ACADS, CX3CR1, GBE1, KRT85*, and *TYR* as possible candidate genes involved in RB oncogenesis, given their association with the retinoic acid pathway (230).

RB is generally described as retinoblastoma predisposition syndrome since germline *RB1* mutations lead to a high risk of second primary malignancies (231). Interestingly, RB onset is reported in 13q deletion syndrome, caused by deletion of part of the long arm of chromosome 13, where *RB1* is located (123, 232) (Table 2). Patients with this syndrome show a very wide phenotypic spectrum depending on the size and the location of the deletion (123, 232, 233).

### Sporadic Retinoblastoma

Sporadic RB is always unilateral. Biallelic loss of *RB1* is found in 98% of cases, whereas 2% show *MYCN* amplification (234, 235). A significant proportion of sporadic RB exhibits somatic mosaicism for *RB1* mutations (236, 237).

#### Uncommon, Moderate-Penetrance and Common, Low-Penetrance Variants

Susceptibility variants have been investigated mostly in patients with hereditary RB. However, given the role of the p53 pathway in RB development, polymorphisms in genes such as *MDM2* (163), *MDM4* (164), and *CDKN1A* (169) could also influence the development of the sporadic form (Table 3). Uncommon variants conferring RB risk may be present in asymptomatic individuals. Indeed, high-throughput analysis revealed that several low-frequency *RB1* variants are present in the human population, including rare alleles disrupting splicing (238).

### Constitutional Chromosomal Abnormalities

Mosaic and non-mosaic chromosomal deletions of 13q14 region are causative of RB (123, 239). Additionally, duplication of 1q21.1, containing the oncogene *BCL9*, has been reported in a patient with bilateral RB (240).

## Wilms Tumor

Wilms tumor (WT), also known as nephroblastoma, is the most common renal malignancy of childhood, with a median age at diagnosis between 2 and 3 years (241) (Figure 2). It is considered an embryonal tumor as it arises from the aberrant kidney development, due to genetic anomalies in genes essential for fetal nephrogenesis (29). WT treatment is successful with a 5-year overall survival of about 90% and 75% for localized and metastatic disease, respectively (82). It is estimated that about 10% of WT cases are caused by genetic predisposition factors, mainly represented by germline pathogenic variants or epigenetic alterations occurring early during embryogenesis (147, 242). The number of known susceptibility loci has significantly increased over the past years, even if our knowledge is still incomplete and further predisposition factors remain to be discovered. The landscape of somatic genetic alterations in WT is quite broad, with classical genetic changes involving *WT1*, the *IGF2* locus, the WNT pathway, *MYCN* and *TP53* but also driver mutations in several additional cancer genes including epigenetic remodelers, miRNA processing genes and transcription factors essential for nephrogenesis (29).

### Familial Wilms Tumor

Several congenital malformation and cancer predisposition syndromes are associated with the risk of developing WT (Table 2). Some of the most known and characterized syndromes are associated with constitutional alterations in *WT1* at 11p13 (60). *WT1* was the first gene identified in WT and encodes a zinc-finger transcription factor, essential for renal and gonadal development (243). A syndrome frequently associated with high risk of developing WT (around 50%) is the Wilms tumor–aniridia syndrome (WAGR), caused by microdeletions of 11p13 including *WT1* and *PAX6* (115, 244). The second *WT1*-related disorder is Denys–Drash syndrome (DDS), due to missense variants in *WT1* exons 8 or 9, which affect critical residues in the zinc finger domains (51). The risk of WT in children with DDS is about 90% (241). Another syndrome, phenotypically similar to DDS but with a lower risk of WT development, is Frasier syndrome (FS), caused by splicing variants that result in an imbalance of *WT1* isoforms (63). The second major WT locus, identified at 11p15 (245), is also characterized by multiple germline epigenetic and genetic changes causing the overgrowth disorder Beckwith–Wiedemann syndrome (BWS). High WT risk is specifically associated with uniparental paternal disomy at 11p15 and to isolated H19 hyper-methylation that results in biallelic expression of *IGF2* and over-activation of the IGF signaling pathway (30, 246). Table 2 reports other constitutional genetic mutations underlying both congenital syndromes and WT predisposition (34, 35, 61, 66, 69, 78, 80, 84, 100, 101, 110, 113).

WT is primarily a non-familial condition, with only about 2% of affected individuals belonging to familial pedigrees (29) (Table 1). A small proportion of familial cases are due to germline *WT1* variants (149, 150) and mutations in the H19 region of 11p15 (151). Two further predisposition loci at 17q21 (*FWT1*) and 19q13 (*FWT2*) were identified by genetic linkage studies, but the causative genes still remain not fully characterized (247). Another cause of familial WT is the presence of inactivating mutations in the *DICER1* miRNA processing gene, also causative of cancer susceptibility in DICER1 syndrome (55). Other recognized familial WT predisposition genes are *CTR9* and *REST* (144, 148, 248). *CTR9* encodes a key component of the PAF1 complex, implicated in maintenance of stem cell pluripotency (144), while *REST* encodes the RE1-silencing transcription repressor, well-known for its role in repressing neural development and differentiation (249). Rare biallelic *TRIP13* mutations have been found in a WES study on familial WT pedigrees (83). *TRIP13* encodes a member of the spindle assembly checkpoint complex, whose inactivation leads to chromosome segregation dysfunction and aneuploidy (83). Pathogenic inactivating mutations of *TRIM28* have been found in about 8% of familial WT in a sequencing study on 890 patients (147). These mutations have been found to show a strong parent-of-origin effect and a robust association with the epithelial subtype of WT (147, 250, 251). The same study reports constitutional mutations in *FBXW7, NYNRIN*, and *CDC73* as contributors to a small number of familial cases, and pathogenic mutations in *TRIM28, FBXW7*, and *KDM3B* as *de novo* events in children with sporadic tumors (147).

It is important to note that, to date, germline pathogenic variants have been identified only in a small proportion of familial WT cases and so that the underlying causative genetic events remain still obscure for the majority of individuals.

### Sporadic Wilms Tumor

Many genetic causes of familial and syndromic WT also contribute to sporadic cases, e.g., constitutional *WT1* mutations and germline 11p15 anomalies (150, 151). It is currently estimated that in sporadic cases the number of predisposition genes is more than 20 (147). Next-generation sequencing (NGS) and GWAS approaches have allowed researchers to discover an ever-growing number of uncommon (Table 1) and common (Table 3) genetic variants associated with WT susceptibility.

#### Uncommon, Moderate-Penetrance Variants

Two recent WGS and WES studies have identified new pathogenic germline variants in *CHEK2* and *PALB2* in children with sporadic WT (131, 132). Both *PALB2* and *CHEK2* are involved in DNA repair pathways and are associated with breast cancer predisposition (62, 252). Germline mutations in *REST* and *TRIM28*, in addition to their role of familial WT predisposition genes, are also responsible for uncommon sporadic cases (148, 251). Additional pathogenic and likely pathogenic variants were identified in predisposition genes such as *TP53, DIS3L2*, and *MLLT1*, but also in candidate genes like *EP300, HDAC4, HACE1, ARID1A, NF1, MYCN*, and *GLI3* (131, 132, 137), that need to be validated in independent cohorts. Finally, exome and transcriptome sequencing studies have revealed constitutional mutations in the miRNA processing genes *DROSHA, DGCR8, DICER1*, and *XPO5* (131, 137), some of which associated with the blastemal subtype of WT (137).

#### Common, Low-Penetrance Variants

The first WT related GWAS study was performed by Turnbull et al. (153), using a dataset of 757 affected and 1.879 controls from North America and subsequently validated in two independent replication series from UK and US populations. They identified two significant SNPs at 2p24 (rs807624 and rs3755132), in the promoter of *DDX1*, and one SNP at 11q14 (rs790356) located near *DLG2*. They also identified candidate predisposition loci at 5q14, 22q12, and Xp22, located near the genes *PCSK9, TCN2*, and *NHS*, which need further validation (153). More recently, the group of Fu and colleagues performed two candidate gene studies on Southern Chinese populations and found a significant association between WT risk and *BARD1* (158) and *KRAS* (171) polymorphisms, respectively. However, both associations need to be validated in larger cohorts.

### Constitutional Chromosomal Abnormalities

Few chromosomal aberrations and copy-number variations (CNVs) are known to be WT predisposing genetic factors. In addition to karyotypic abnormalities affecting 11p13 and 11p15 (60), a very small number of WT patients with gain of entire chromosomes have been reported, specifically with trisomy 18 and trisomy 13 (60). Rare chromosomal aberrations have been identified at 2q (60, 253, 254) and 7q (255, 256) regions, with terminal deletions and balanced and unbalanced translocations. A constitutional *de novo* balanced translocation was also identified in a child with bilateral WT, affecting the tumor-suppressor gene *HACE1*, also reported as NB susceptibility gene. *HACE1* controls growth and apoptosis and is often somatically mutated in WT (257). Moreover, gain of *MYCN* (2p24), which is predominantly a somatic event, has been reported as a rare germline aberration (258). Finally, in 2020, a germline duplication of *SUZ12* has been detected in a WT patient carrying other germline pathogenic variants in new candidate cancer predisposition genes (3).

## Osteosarcoma

Osteosarcoma (OS) is the most common primary bone cancer. This tumor has a bimodal distribution with a high peak during adolescence and a smaller peak in elderly individuals (259) (Figure 2). Survival rates for children and young adults with non-metastatic disease have remained at 60–70%; however, outcome is reduced in patients with metastases (259). Unlike other childhood sarcomas, which are characterized by specific chromosome rearrangements and low mutation rate, complex genomic rearrangements are involved in OS. Indeed, OS exhibits extensive intra-tumoral heterogeneity and has a higher mutation rate (259).

### Familial Osteosarcoma

OS is a sentinel cancer in many heritable cancer predisposition syndromes, including autosomal dominant cancer predisposition syndromes such as LFS (73–75) and Diamond–Blackfan anemia (47–50) (Table 2). Furthermore, recessive cancer syndromes associated with OS are Rothmund–Thomson syndrome (102–105), Baller–Gerold syndrome (20, 21), RAPADILINO syndrome (106, 107), Werner syndrome (118–120), Bloom syndrome (32), and ATR-X syndrome (19). OS has also been seen to arise in Paget's disease of bone (98, 99).

### Sporadic Osteosarcoma

Targeted gene sequencing and WGS and WES studies have identified uncommon variants in tumor-suppressor and cancer predisposition genes (Table 1), while candidate gene, pathway studies, and GWAS have discovered common variants in genes involved in several key pathways for OS development (259) (Table 3).

#### Uncommon, Moderate-Penetrance Variants

In 2015, a sequencing study on 765 germline DNA samples showed the presence of uncommon *TP53* germline variants that could contribute to OS development; 3.8% of these variants were associated with LFS, and 5.7% were uncommon exonic variants of uncertain clinical significance (138). Another sequencing study on 1120 cases found 7/39 OS patients carrying pathogenic and likely pathogenic variants in *TP53, RB1, APC, MSH2*, and *PALB2* (2). In 2016, a targeted exon sequencing on 1162 patients with sarcoma found that >50% of all patients carried pathogenic variants in *TP53, BRCA2, ATM, ATR*, and in *ERCC2* (128). Among 11% of patients with OS, one patient showed a probable pathogenic variant in *ERCC2*. In the same work, an excess of functionally pathogenic variants in *ERCC2* was found to enhance cell sensitivity to cisplatin, commonly used in the treatment of OS (128). Recently, a sequencing study of 1244 OS patients showed that 28% of patients carried pathogenic and likely pathogenic variants in OS susceptibility genes, identifying new candidates (*CDKN2A, MEN1, VHL, POT1*, and *ATRX*) that require further confirmation in independent cohorts (129).

#### Common, Low-Penetrance Variants

In 2013, the first GWAS study on 941 cases and 3291 controls of European ancestry, identified two risk loci, one at 6p21.3 (rs1906953) mapping in intron 7 of *GRM4*, and the other at 2p25.2 (rs7591996) in an intergenic region (173). Subsequently, a GWAS study on OS metastasis at diagnosis identified rs7034162 at 9p24.1 (in *NFIB*) associated with metastasis (176). Functional investigations showed that reduced *NFIB* expression, due to the risk allele of the rs7034162 SNP, promoted an increase of OS cell migration, proliferation, and colony formation (176). In 2016, a case–control study identified that, for SNPs in genes associated with inter-individual variation in leukocyte telomere length (LTL) (*ACYP2, TERC, NAF1, TERT, OBFC1, CTC1*, and *RTEL1*), the allele associated with longer LTL increased OS risk, mainly rs9420907 in *OBFC1* (165). These findings were confirmed in 537 OS cases belonging to California Cancer Registry (166).

### Constitutional Chromosomal Abnormalities

Next to the heterogeneous somatic CNV scenario present in OS, in a study conducted on 54 patients with childhood tumor, two large germinal CNVs were identified in 2 OS patients: dup4q13.33 of 476 kb containing *STATH, CSN1S2B, CABS1, CSN1S1, CSN2, HTN3, HTN1, CSN1S2A, C4orf40, ODAM, FDCSP*, and *CSN3*; and dup18q21.33 of 600 kb containing *RNF152, CDH20*, and *PIGN* (240). In 2020, a duplication of *DDX10* in an OS patient with a germline variant in *GJB2* has been reported (3).

## Rhabdomyosarcoma

Rhabdomyosarcoma (RMS) is the most common soft tissue sarcoma in childhood and represents a high-grade neoplasm of skeletal myoblast-like cells. Currently, 5-year overall survival of pediatric RMS exceeds 70% (260). The two major histological subtypes are embryonal (ERMS, 67%) and alveolar (ARMS, 32%) (261). ARMS is uniformly distributed among the different age groups (Figure 2) and has a worse prognosis; ERMS has a bimodal distribution (the first peak in early childhood and the second one in early adolescence) and has a better outcome (260, 262) (Figure 2). At somatic level, ARMS is often associated with fusion of *FOXO* and *PAX3* or *PAX7*, while ERMS does not show such translocations, but it is characterized by loss of heterozygosity at 11p15.5 as well as mutations in *TP53, NRAS, KRAS, HRAS, PIK3CA, CTNNB1*, and *FGFR4* (263). Since a small but substantial fraction of ARMS patients do not harbor one of these translocations, and tumors from those patients are biologically and clinically similar to ERMS, the disease classification has been further refined dividing RMS into “fusion-positive” RMS (FPRMS) and “fusion-negative” RMS (FNRMS) subtypes.

### Familial Rhabdomyosarcoma

Although RMS is primarily sporadic (264, 265), it arises in several syndromes. Cancer predisposition syndromes appear to be more frequent in patients with ERMS than in those with ARMS (260). Among syndromes commonly associated with RMS and reported in Table 2 (24–27, 42, 43, 52–54, 64, 75, 80, 81, 86, 87, 89–92, 94, 96), a high RMS risk is associated with RASopathies-like type I neurofibromatosis (NF1) (deletions in *NF1*), Costello syndrome (*HRAS* mutations), and Noonan syndrome (germline variants activating RAS-MAPK pathway), highlighting the tight dependence of RMS on the RAS pathway, which results to be activated in 40% of sporadic ERMS (263, 266, 267). In particular, up to 25% of children affected by Costello syndrome shows high RMS risk (43, 268). In addition, children who have a first-degree relative with cancer, particularly if the cancer occurred at a young age (<30 years), show an increase in RMS risk, especially of ERMS (269).

### Sporadic Rhabdomyosarcoma

Unlike OS and Ewing sarcoma, GWAS studies for RMS have not been published (260) and few studies identified uncommon germline variants associated with tumor susceptibility (2, 52, 133, 139, 142, 270) (Table 1).

Many studies have found the presence of *DICER1* germline mutations in sporadic RMS patients for whom DICER syndrome has been ruled out (52, 142). WES and WGS on 1,120 patients with pediatric cancers identified germline pathogenic variants in 3/43 RMS patients in *TP53* and *BRCA2* (2). In a cohort of 66 patients with sarcoma, one patient with ARMS showed a protein-truncating variant (in *ERCC4*) co-occurring with predicted pathogenic mutations (in *ATM, FANCI*, and *MSH6*), suggesting a possible collective impact of these genetic variants on DNA repair and genomic instability, therefore conferring susceptibility to tumorigenesis (133).

## Ewing Sarcoma

Ewing sarcoma (EWS) is the second most frequent primary skeletal tumor that mainly affects bone and can also arise in soft tissue. It occurs in children, adolescents, and young adult (Figure 2). It is highly aggressive, with a survival of 70–80% for patients with standard-risk and localized disease and 30% for those with metastasis at diagnosis (20–25% of those resistant to intensive therapy) (271). EWS is characterized by low somatic mutation rate (272–274), mainly including fusions between *EWSR1* and members of the *ETS* gene family, usually *EWSR1-FLI1*, that play a key role in its pathogenesis. The chimeric protein EWSR1-FLI1 leads to the production of an oncogenic transcription factor that binds GGAA motifs (174, 271, 275, 276).

### Familial Ewing Sarcoma

To date, no susceptibility genes to familial forms of EWS have been reported, and only case reports about siblings and cousins affected by this tumor have been documented (277, 278). On the basis of these isolated clinical cases, the presence of other cancer types among familial members of EWS patients (279, 280) suggests an important contribution of genetic susceptibility factors in this tumor. Nowadays, EWS is not considered part of predisposition syndromes because of its rare occurrence among these (281).

### Sporadic Ewing Sarcoma

WES, WGS, and GWAS studies have led to the identification of uncommon (Table 1) and common (Table 3) germline variants associated with the risk of developing EWS. Despite the rarity and the paucity of information about familial cases, most of the known genetic scenario on this tumor concerns the sporadic form.

#### Uncommon, Moderate-Penetrance Variants

Two WGS and WES studies on EWS revealed an over-representation of uncommon pathogenic and likely pathogenic variants in DNA repair and cancer-predisposing syndrome genes (2, 130). Studies on small cohorts of patients identified other uncommon germline variants in *BRCA2* (146) and in *PTPRD* (140).

#### Common, Low-Penetrance Variants

In 2012, the first GWAS on EWS found 3 susceptibility loci at 1p36.22, 10q21, and 15q15, identifying a strong association of EWS risk with rs9430161 (25 kb upstream of *TARDBP*) and rs224278 (5 kb upstream of *EGR2*), and a modest association with rs4924410 (at 15q15) (172). The second GWAS detected a tagging variant strongly associated with EWS at 15q.15.1 (rs2412476 near *BMF*) and new risk loci at 6p25.1, 20p11.22, and 20p11.23 (152). Expression quantitative locus (eQTL) analyses identified candidate genes at 6p25.1 (*RREB1*) and 20p11.23 (*KIZ*) (152). Independent studies showed that a different number of germline GGAA repeats in polymorphic enhancer-like GGAA microsatellites impacts the binding between these regulatory elements and EWS cancer driver mutations (*EWSR1-FLI1*), affecting downstream genes expression (174, 178, 282).

These studies further suggest that cooperation between regulatory germline variants and somatic mutations can drive oncogenesis and create a major source of inter-tumor heterogeneity, determining clinical outcome and drug response through modulation of a druggable key downstream player.

### Constitutional Chromosomal Abnormalities

Only one study reports the presence of germline CNV associated with EWS, describing a 14-year-old male with EWS carrying an intragenic deletion in *PTPRD* (283). Notably, germline and somatic variants in *PTPRD* have been already identified in a limited number of EWS patients (140).

## Conclusions

**Figure 3:**
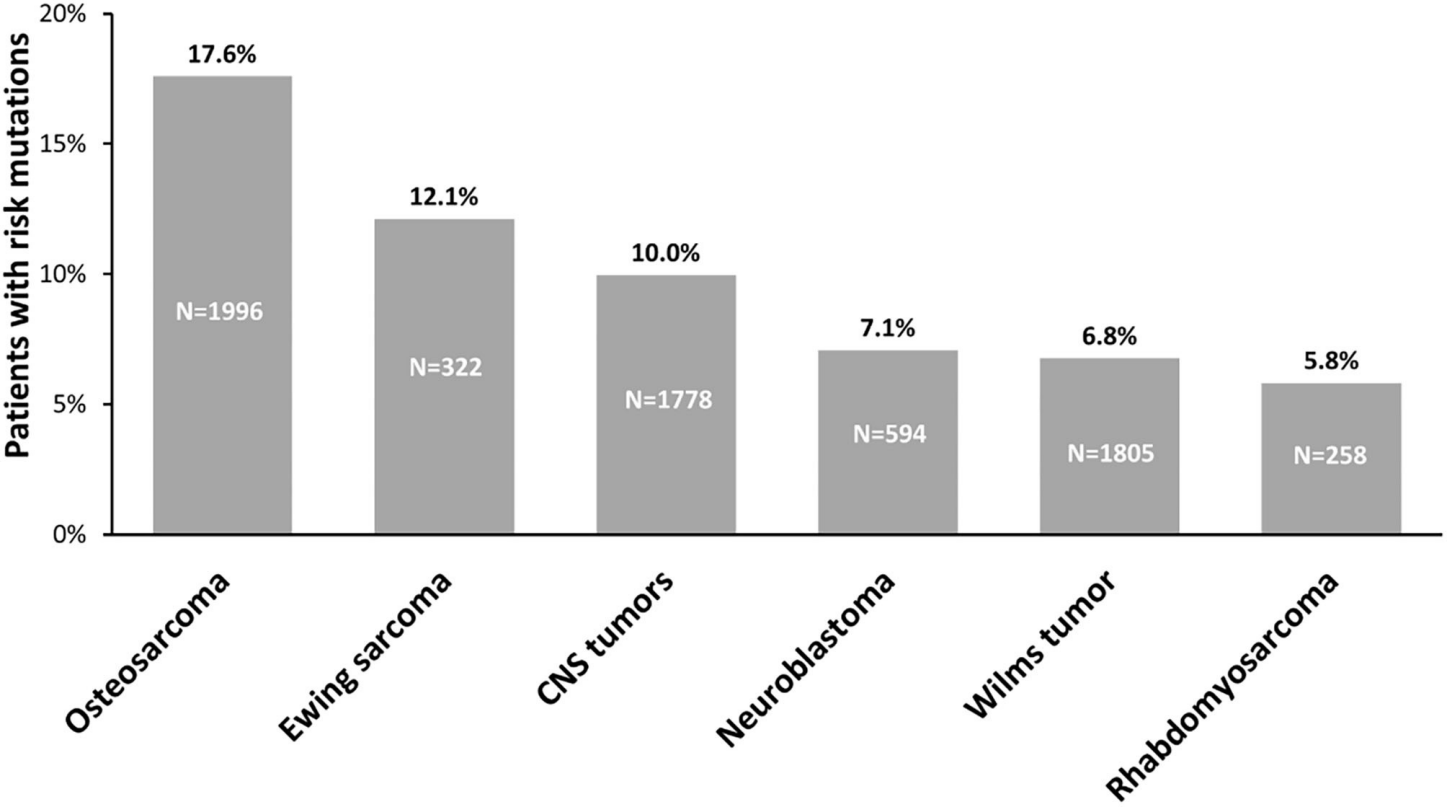
Prevalence of germline predisposition in pediatric tumors. The percentage of germline predisposition due to uncommon, moderate-penetrance variants, reported above the bars, has been calculated evaluating the number of patients carrying pathogenic and likely pathogenic variants on the total number of patients from the cohorts analyzed for each tumor: CNS tumors: (3, 15, 17); neuroblastoma: (2, 3, 135, 146, 196, 197, 200); Wilms tumor: (3, 131, 132, 137, 148, 150, 251); osteosarcoma: (2, 3, 129, 138); rhabdomyosarcoma: (2, 3, 52, 139); Ewing sarcoma: (2, 3, 130, 146). N, number of patients analyzed in cohorts; CNS, central nervous system.

For a long time, the prevalence of childhood cancer attributed to genetic predisposition was generally considered very low. However, to date, WGS, WES, and GWAS studies performed on pediatric cancers have made it possible to highlight a strong contribution of germline variants to tumorigenesis, helping us to better understand the etiology underlying pediatric tumors. Indeed, an important body of work allows us to highlight that the prevalence of hereditable risk variants in pediatric solid cancers ranges between 6% and 18% (Figure 3). These variants generally affect the functions of genes belonging to biological processes linked to tumorigenesis, such as cell-cycle control, apoptosis, DNA repair, and transcriptional regulatory programs. The enrichment of genetic alterations in these pathways is often due to a bias because, since germline variant analysis is a highly challenging task in general, the vast majority of studies are based on a “candidate-gene” approach, which means they focus on specific subsets of genes already known to play a key role in cancer predisposition and tumorigenesis. For this reason, it may be useful exploiting a genome-wide scale approach, e.g., exome-wide association studies, to investigate the presence of genetic alterations predisposing to cancer also in genes involved in pathways others than the ones above mentioned. This approach may contribute in a meaningful way to the current knowledge of the mechanisms underlying solid pediatric tumors onset.

A very recent study reports a high number of germline variants in new candidate susceptibility genes, highlighting that some of them carry druggable alterations (3). It should be emphasized that the presence of germline variants in target therapeutic genes could improve current approaches of personalized therapy, making them more efficient and less toxic to patients. Furthermore, a more in-depth investigation of the germline component underlying tumor development should also be performed on pediatric solid tumors for which there is not yet a broad knowledge of germline landscape (e.g., thyroid carcinoma, melanoma) (284–289).

Our literature review reveals that the presence of specific germline mutations is often associated with increased frequency of somatically acquired cancer-specific abnormalities (such as aberrations, rearrangements). The interplay between somatic and germline mutations may be at the basis of high interindividual tumor heterogeneity (290). For example, the cooperation between regulatory germline variants and somatic mutations underlines the importance of regulatory regions to stratify patients into risk groups to predict the clinical outcome and therapeutic approaches (290). In NB, inherited deleterious variants in genes that code for proteins involved in chromosomal segregation, centrosome segregation, DNA repair, and spindle apparatus machinery are thought to be the cause of chromosome instability at somatic levels (199). A similar germline–somatic interaction has been proposed for MB; indeed, germline *TP53* mutations are often found in combination with tumors characterized by catastrophic DNA chromothripsis. Determining if germline risk alleles predispose to genomic instability in pediatric cancers is an important research objective for biologists and geneticists. Another interesting research field is related to the impact of risk alleles on genomic regions that regulate mutated cancer driver genes. The mechanisms underlying this type of interaction between germline–somatic variation have been elegantly elucidated in the EWS (174, 178, 282), and it is reasonable to think that it is common to other pediatric tumors as well. No relevant study has investigated the possible interplay between germline variations and epigenetic somatic events. For instance, there is an urgent need to find possible associations between germline risk alleles and DNA methylation of tumor. Studies integrating information on germline, somatic, and epigenomic variations using gene expression data as the intermediate phenotype may unravel the biological mechanisms underlying oncogenic interactions and cooperation of these different types of genomic variations.

The low number of recurrent somatic mutations in some pediatric cancers, compared to adult ones (135), does not explain the clinical heterogeneity and the resulting need for personalized therapies in tumors. Confirming a germline contribution to the clinical heterogeneity, some studies have highlighted that specific pathogenic variants are much more common in specific tumor histotypes (137, 147) and these associations could be used for the management and stratification of patients. Thereby, implementing screening tests with the introduction of germline detection would bring clinical benefits. In addition, screening for germline and somatic components of the tumor could lead to the identification of new prognostic markers to monitor cancer and predict clinical outcome. Finally, the use of these information in screening tests is important in the context of genetic counseling, to monitor and supervise family members of patients.

It is also important to note that many genetic syndromes such as Beckwith–Wiedemann, Costello, Fanconi anemia, Gorlin, Noonan syndrome, Li-Fraumeni, and others (Table 2) are both characterized by genetic and/or allelic heterogeneity and associated with the risk to develop different types of pediatric cancers. Therefore, NGS-based cancer gene panel tests should be performed in children with a genetic syndrome to ensure the patient a more precise diagnosis and to be able to assess the risk of developing a cancer disease. A clinical management that includes a cancer genetic test not only is useful to indicate a modification of the surveillance that also integrates periodic and cancer specific diagnostic tests, but over time it will increase our knowledge of genetic risk variants and thus will give a clearer picture of cancer risk in children affected by genetic syndrome. This surely can have a positive impact on improving patient care and survival.

## Author Contributions

MC and AI contributed to the design, reviewing, and editing of this manuscript. AM, SC, TM, and MT contributed to the design, writing, and editing of this manuscript. All authors have read and agreed to the published version of the manuscript.

## Conflict of Interest

All authors were employed by the company CEINGE Biotecnologie Avanzate.
